# Primary disseminated intraabdominal hydatidosis: a case report

**DOI:** 10.1186/s13256-022-03262-5

**Published:** 2022-01-28

**Authors:** Maryam Fasihi Karami, Amin Bahreini, Abdollah Rafiei, Ali Asghar Dastyar, Molouk Beiromvand

**Affiliations:** 1 Department of Parasitology, School of Medicine, Ahvaz Jundishapur University of Medical Sciences, P.O. Box 61357-15794, Ahvaz, Khuzestan Iran; 2 Department of Surgery, Ahvaz Jundishapur University of Medical Sciences, Ahvaz, Iran

**Keywords:** Hydatidosis, Intraabdominal hydatidosis, *Echinococcus granulosus*, Cystic echinococcosis, Iran

## Abstract

**Background:**

Hydatidosis, a zoonotic disease caused by the larvae of *Echinococcus granulosus* sensu lato (*E. granulosus* s.l.), can be primary or secondary. However, primary disseminated intraabdominal hydatidosis is a rare form of the disease, accounting for about 2% of all intraabdominal cysts.

**Case presentation:**

We report herein a case of primary disseminated intraabdominal hydatidosis with multiple organ involvement in a 51-year-old Iranian man presenting to a healthcare facility with abdominal pain. During the physical examination, two abdominal masses were palpated. Ultrasound and computed tomography revealed six cystic lesions in the patient’s liver, subhepatic region, pelvic, and omentum. Afterward, he underwent surgery, during which the cystic lesions were completely removed. The patient received albendazole (400 mg/kg/day) postoperatively and was recommended to continue the treatment for 4 months.

**Conclusions:**

Although primary disseminated intraabdominal hydatidosis is rare, this problem is of great importance due to the fertility of cysts and the high risk of recurrence. Therefore, it is recommended to follow such patients with imaging modalities and enzyme-linked immunosorbent assay for native antigen B (AgB). In addition, patients should undergo albendazole therapy postoperatively for 4 months.

## Background

Hydatidosis (cystic echinococcosis) is a zoonotic disease caused by the larvae of *Echinococcus granulosus* sensu lato (*E. granulosus* s.l.). Canids and ungulates are the definitive and intermediate hosts in the life cycle of *E. granulosus*, respectively. However, human is an accidental intermediate host [1]. The organs commonly affected by this disease include the liver (70%) and lungs (20%), while involvement of other organs is rare [2]. However, intraabdominal hydatidosis can be observed in other organs such as the peritoneum, spleen, kidney, and pancreas [3]. Also, hydatidosis can be primary or secondary [4], with primary disease occurring accidentally due to ingestion of *E. granulosus* eggs [2]. However, in secondary form, cysts develop by rupture of primary cysts due to operation, trauma, or other agents [4]. Several previous studies have reported that peritoneal hydatidosis is usually caused by daughter cysts (secondary cysts) [5, 6]. Therefore, primary peritoneal hydatidosis is rare and accounts for only 2% of cases of abdominal hydatidosis [7]. We reported herein a rare case of primary disseminated intraabdominal hydatidosis.

## Case presentation

**Fig. 1:**
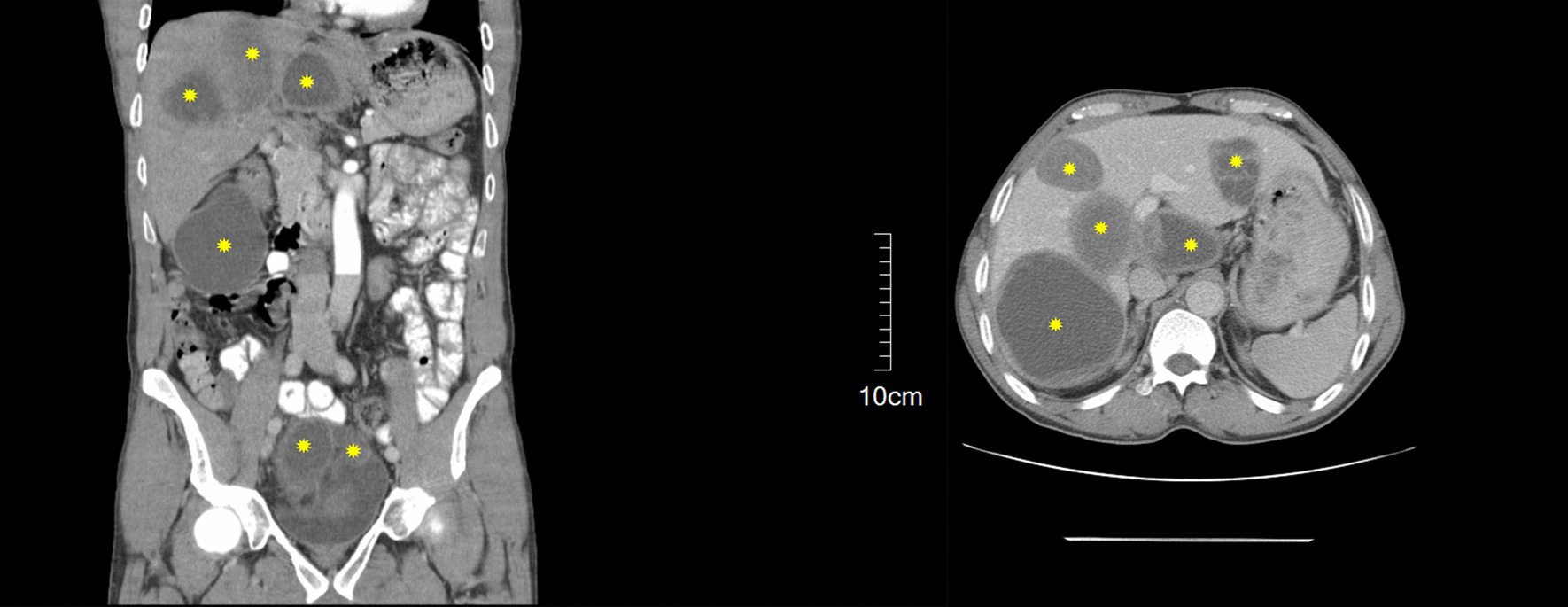
Abdominal CT scan showing multiple cystic lesions in the liver, subhepatic region, omentum, and pelvis (yellow stars)

**Fig. 2:**
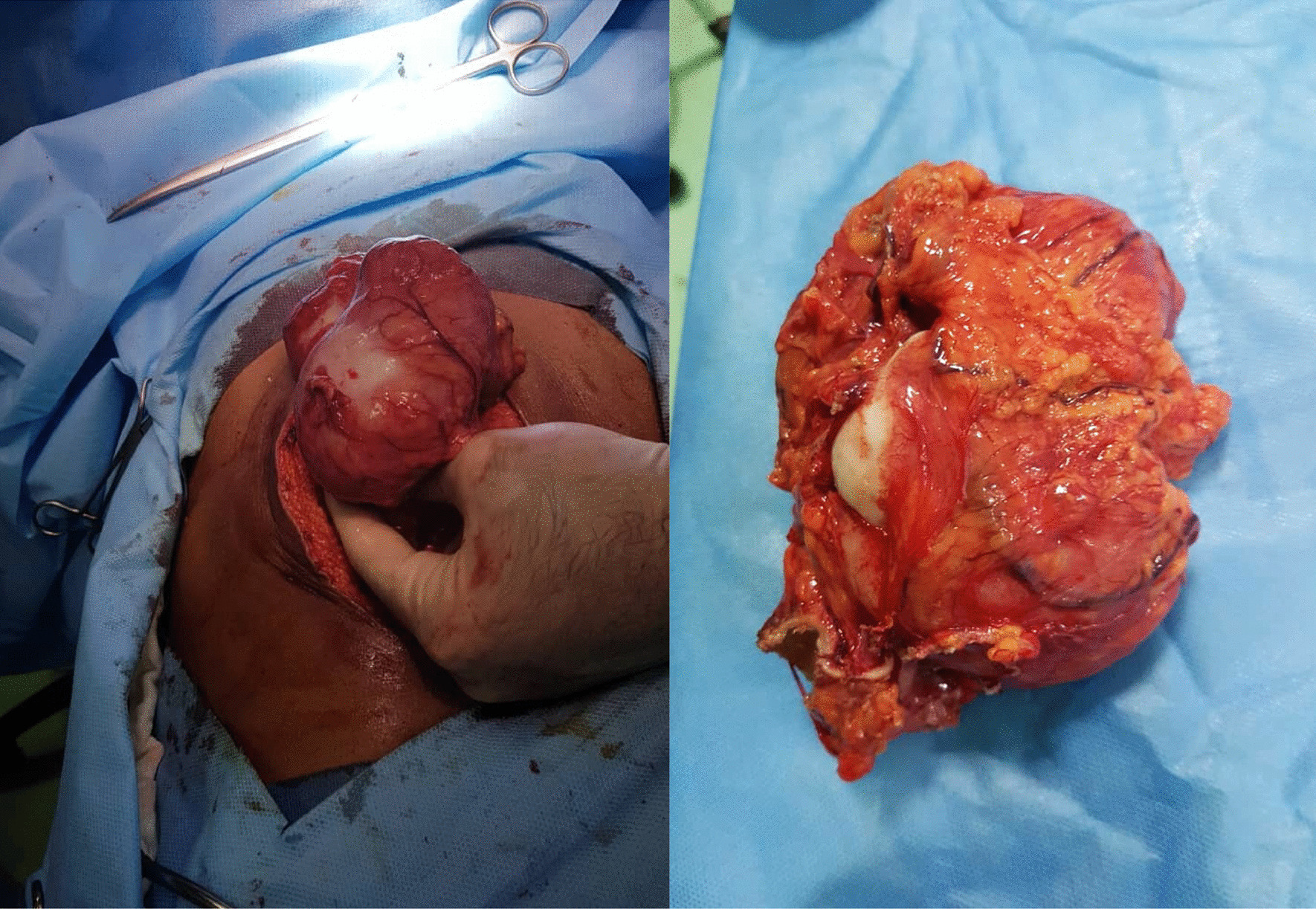
Intraoperative photos of surgical field

A 51-year-old Iranian man who was a farmer residing in a rural region in the southwest of Iran presented to our surgery unit with history of recent abdominal pain. The patient had no history of previous surgery for hydatid cyst excision. Moreover, he had the following vital signs on physical examination: body temperature of 36.5 °C and blood pressure of 120/60 mmHg. Also, two abdominal masses were palpated during abdominal examination. Laboratory investigations revealed hemoglobin of 11.9 g/dL and white blood cell (WBC) count of 10.4 × 10^3^/µL, including 68% neutrophils and 32% lymphocytes. Also, the patient underwent enzyme-linked immunosorbent assay (ELISA) for native antigen B (AgB), which was positive. Abdominal ultrasound and CT scan revealed six cystic lesions in the patient's abdomen, including two in the right hepatic lobe, one in the left hepatic lobe, one in the subhepatic region, one in the pelvic, and one in the omentum (Fig. 1). Following general anesthesia, a midline abdominal incision was made, and the abdomen was explored. The hydatid cyst on the omentum was resected by Harmonic scalpel, while the subhepatic cyst attached to the gallbladder was completely drained. Also, the one in the pelvic area was gently drained while checking the urinary bladder and rectum after packing the surrounding area with several sponges soaked in 0.5% silver nitrate. Subsequently, the germinal layer of the cyst was removed, and a Jackson–Pratt (JP) drain was inserted into the pelvis. Afterward, the cyst in the left hepatic lobe was drained, and a JP drain was inserted into the left hepatic lobe after suturing the bile duct. Finally, the two cysts in the right hepatic lobe were fully drained as well, and a JP drain was placed in the right hepatic lobe (Fig. 2).

**Fig. 3:**
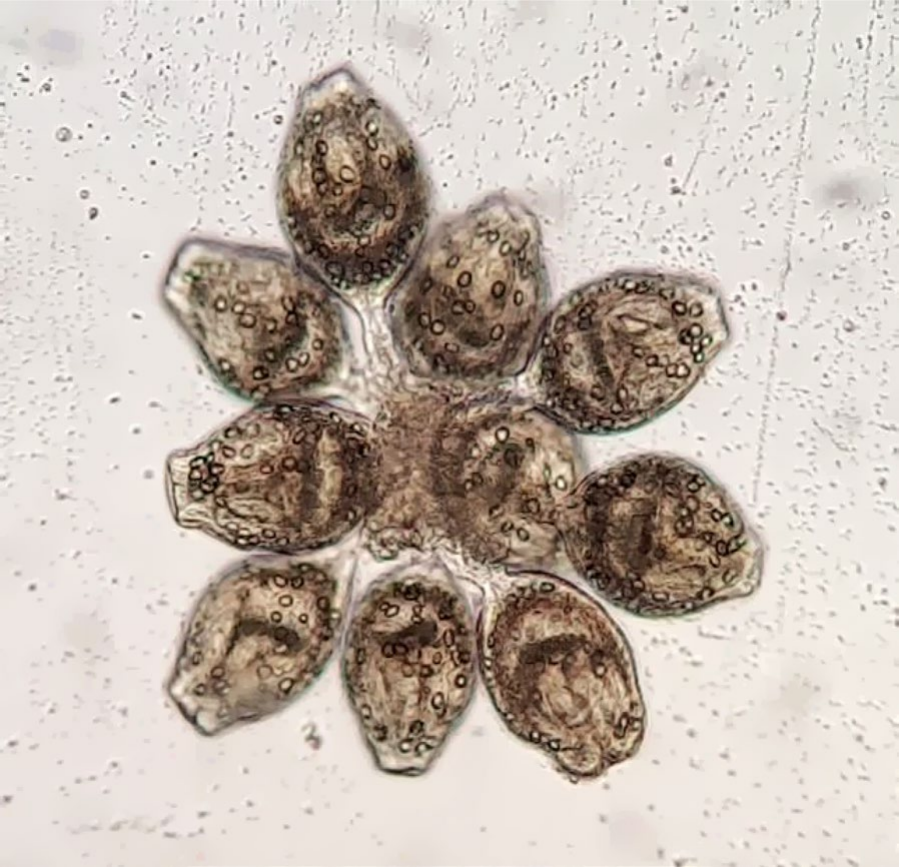
Cluster of protoscoleces of *E. granulosus* collected from cyst fluid

Examination of a direct smear of fluid from the hydatid cysts revealed brood capsules and protoscoleces of *E. granulosus* (Fig. 3). In addition, histological examination of the cystic lesions confirmed hydatidosis. Therefore, the patient underwent albendazole therapy (400 mg/kg/day) for 4 months and was asked to return for follow-up 4 months after discharge until 2 years later.

## Discussion and conclusions

Hydatidosis, a zoonotic disease caused by larvae of *E. granulosus* s.l., *E. ortleppi*, *E. equinus*, and *E. canadensis*, has a cosmopolitan distribution, with an estimated annual incidence above 50 cases per 100,000 people in the human population of endemic areas [8]. Moreover, the disease has an economic burden of 3 billion dollars annually, including both treatment and livestock loss [9]. Although hydatidosis usually affects the liver and lungs, it can involve other organs, albeit less frequently [4]. The cystic lesions of the disease can be primary or secondary [4], while as a rare form of primary hydatidosis, disseminated intraabdominal hydatidosis accounts for about 2% of all intraabdominal cases [10]. Such dissemination may occur through either lymphatic or systemic circulation [11].

The disease is usually diagnosed on the basis of serological and imaging techniques [12]. The World Health Organization Informal Working Group on Echinococcosis (WHO-IWGE) has classified cystic echinococcosis (CE) into three types based on ultrasound findings: active (CE1 and CE2), transitional (CE3), and inactive (CE4 and CE5). Types CE1 and CE2 are usually fertile, but CE3 may include daughter cysts that usually start to degenerate. In addition, most CE4 and CE5 cases are not fertile [13]. Since most CE1 and CE2 cysts are fertile and contain daughter cysts, their rupture during surgery or trauma can lead to disseminated disease in the peritoneum [10, 14]. Therefore, scoliotic agents, such as 0.5 silver nitrate, hypertonic saline, and 10 formaldehyde, are typically used during surgery to prevent formation of secondary cysts. However, some hydatid cyst cases can be eradicated by repeated surgeries over several years [15].

Most intraabdominal hydatid cysts are asymptomatic because they grow quite slowly [3]. Some have nonspecific manifestations such as abdominal pain, dyspepsia, anorexia, and vomiting [16]. Despite the extensive abdominal involvement in both lobes of the liver, the subhepatic space, omentum, and the pelvic, the present case only had abdominal pain. Symptoms of hydatidosis mostly depend on the cyst’s size, location, and type and whether it is complicated or not [3]. Previous studies have reported that abdominal pain, specifically in the right upper quadrant, is the most common symptom of affected patients [3, 17, 18].

Although disseminated intraabdominal hydatidosis is rare, its management is highly important. In the present case, microscopic examination showed that the excised cysts were fertile. Given the multiple organ involvement, the fertility of the cysts, and their possibility of rupture or leakage during surgery, the risk of secondary cyst development and disease recurrence is increased. Therefore, it is recommended to follow up the patient using imaging modalities and ELISA. Also, albendazole therapy should be started and continued for 4 months postoperatively.

## Data Availability

All data generated or analyzed during the present study are included in the paper.

## Abbreviations

CE: Cystic echinococcosis
*E. granulosus*: *Echinococcus granulosus*
US: Ultrasound
CT: Computed tomography
ELISA: Enzyme-linked immunosorbent assay
AgB: Antigen B
WBC: White blood cell
WHO-IWGE: World Health Organization Informal Working Group on Echinococcosis
